# The self-correction and influence factors of congenital auricular deformity: A prospective cohort study

**DOI:** 10.1371/journal.pone.0309621

**Published:** 2024-10-07

**Authors:** Jincheng Huang, Kun Zou, Min Yang, Yanjun Fan, Jinjie Xia, Li Zhao

**Affiliations:** 1 Chengdu Center for Disease Control and Prevention, Chengdu, Sichuan province, China; 2 West China Second Hospital of Sichuan University, Chengdu, Sichuan province, China; 3 Faculty of Health, Art and Design, Swinburne Technology University, Melbourne, Australia; 4 National Center for Women and Children’s Health, China Center for Disease Control and Prevention, Beijing, China; 5 Department of Health Policy and Management, West China School of Public Health and West China Fourth Hospital, Chengdu, Sichuan province, China; Universiti Sains Malaysia, MALAYSIA

## Abstract

**Objective:**

To prospectively observe the self-correction of congenital auricular deformity (CAD) and explore the potential factors affecting the self-correction.

**Methods:**

This study was a multi-center prospective observational study. Newborns aged 0–3 days from 12 Maternal and Child Health Hospitals or Maternity Hospitals were chosen as the participants and prospectively followed up until week 6 after birth. The primary and secondary outcome was the score of deformity, and the secondary outcome was the improvement rate, respectively.

**Results:**

A total of 135 newborns diagnosed with CAD (237 ears) were recruited. Boys and girls accounted for 50.37% (117 ears) and 49.63% (120 ears). The top morphological type was the constricted ear (107 ears, 45.15%). The score of deformity at baseline, week 3, and week 6 after enrollment was 4.00, 3.00, and 2.00, decreasing over time (P < 0.05). The higher the severity of deformity, the worse the self-correcting effect (P < 0.05). The scores of deformity of Stahl’s ear were lower than those of others after follow-up (P < 0.05). No significant differences among the scores of deformity in different genders (P >0.05). The total improvement rate at week 3 and week 6 was 29.96% (71/237 ears) and 37.13% (88/237), respectively. The improvement rate of the Stahl’s ear at week 3 and week 6 after enrollment was higher than that of four other morphological types (P < 0.05).

**Conclusions:**

Some CAD tends to self-correction, but for most CADs, there is still a need for early correction. Morphological types and severity of deformity are the main influencing factors on self-correcting effect, whereas sex was not.

## Background

In addition to structural deformity, congenital ear malformation also includes morphological deformity, which is caused by abnormal muscle development or abnormal external forces, without obvious deficiencies in cartilage volume, and is known as congenital auricular deformity (CAD) [1,2]. CAD has multiple morphological types, and there is no uniformity in the classification criteria among different scholars [3,4]. Byrd classified CAD into nine types of ear: prominent ear, constricted ear, cup ear, lop ear, hidden ear, Stahl’s ear, helical rim deformity, conchal crus and compound deformity. Most previous studies adopted this typing approach [1]. In 2019, the Pediatric Group of the Chinese Medical Association, Branch of Otolaryngology, Head and Neck Surgery, formulated and promulgated the Expert Consensus on Techniques for the Correction of Earmolds of Congenital Auricular Deformities (abbreviated as the "Expert Consensus") [5]. CAD was classified into seven types: cup ear/lop ear, conchal crus, prominent ear, hidden ear, Stahl’s ear, helical rim deformity and conchal adhesion. At the same time, the Expert Consensus considered that cup ear and lop ear belonged to the category of constricted ear.

The incidence of CAD in newborns has been reported abroad to range from 55.2% to 57.5% [1,6]. A study by domestic scholar Wu in 2013 pointed out that the incidence of neonatal CAD in the Pearl River Delta Region was 43.46% [7]. At the same time, the prevalence of different morphological types of CAD varied considerably between countries and between regions of the same country [8,9]. CAD not only affects the appearance of the child, but in severe cases, hearing loss may occur, which is detrimental to the physical and mental health development of the child. However, CAD has a certain tendency to self-heal. Matsuo observed this phenomenon in the 1990s and noted that lop ear and Stahl’s ear recovered themselves better [6]. Nevertheless, some later scholars have proposed a different view, such as Smith’s study revealing that most CAD failed to self-heal [10]. Similarly, Merlob did not observe a tendency for CAD to self-heal [11]. It can be seen that the conclusion of self-healing in CAD has been viewed differently in different studies and the conditions for self-healing are unknown.

Some studies tried to explore the factors that influence the self-healing of CAD. Wang et al. pointed out that Stahl’s ear had the highest rate of self-correction at 1 month after birth, and mode of delivery had no effect on the rate self-correction [12]. Kim et al. followed up 43 children for up to a year, noting that prominent and cup ears had low rate of self-correction [13]. It can be seen that previous studies have mainly analysed the effect of morphological typing on CAD self-correction and less on other factors.

In recent years, non-invasive orthodontics for CAD has grown rapidly in clinical practice, benefiting an increasing number of children. However, given the existence of self-healing in CAD, the need and timing of non-invasive correction still require more support from evidence-based evidence. Otherwise, there may be a waste of medical resources due to premature intervention, or the best time to intervene may be missed due to wrong judgment.

Overall, there were few studies about the self-correction and its influencing factors of CAD in China and abroad, and most of the existing research was single-center or hospital studies, making it difficult to avoid selection bias. Based on previous research, the study hypothesis is that that the self-correction of CAD may be influenced by factors such as morphological type, gender and disease severity. Therefore, this study recruited 0–3 day-old newborns with CAD at 12 medical institutions in China and prospectively observed the self-correction and potential influencing factors of CAD in the short term, providing a testimonial basis for the precise prevention and treatment of this type of malformation.

## Methods

### Study design and participant

This study was a multi-center prospective observational study. 12 Maternal and Child Health Hospitals or Maternity Hospitals in Southwest, Central, South, East, North, and Northeast China were chosen as sub-centers during the period of January 20, 2021 to May 30, 2021.

Newborns aged 0–3 days were chosen as the participants. Inclusion criteria: (1) Newborns 0–3 days old. (2) Their parents informed and signed written consent. (3) Diagnosis of CAD in one or both ears, including cup ear, lop ear, conchal crus, prominent ear, hidden ear, Stahl’s ear, helical rim deformity, conchal adhesion, compound deformity, and other types. The diagnosis and morphological types of CAD were based on the Expert Consensus [5] and Byrd’s study [1].

Exclusion criteria: (1) Preterm infants (gestational age < 37 weeks). (2) The presence of congenital hearing impairment. (3) Concomitant multi-organ malformation. (4) Those with other health-related reasons for not being suitable for the study. (5)Those whose parents wanted to wear ear molds early.

This study was a sub-project under the "Study on the prevention and treatment mode of congenital auricular deformity based on RCT", which was approved by the Medical Ethics Committee of Sichuan University (Ethical approval number: K2020016).

### Sample size

The value of *π* was set as 51.56% [14], and that of δ was 0.2*π. The lost to response rate was 20%, and the minimum sample size required for our study was 108 according to the relevant formula below.


$$n={\left(\frac{Z_{\alpha /2}}{\delta }\right)}^{2}\pi (1-\pi )$$

### Measurement and procedure

Continuous screening of newborns born 0–3 days at our institution was performed by a trained and qualified obstetrician or neonatology physician or otolaryngologist at each sub-center [15], and newborns who met the inclusion criteria were recruited. After obtaining consent from the newborn’s parents and signing the informed consent form, the following baseline information was collected by Case report form (CRF): (1) Basic demographic and delivery information of newborn, such as sex, birth length, birth weight, mode of delivery, week of gestation. (2) Basic demographic of parents. (3) Type and severity score of deformity of CAD, and photographs of both auricles were taken.

A score of deformity: The severity of the deformity was evaluated by trained and qualified project members of the sub-center via the visual analog scale (VAS) at weeks 3 and 6. The score range was 0 to 10, where 0 meant no deformity and 10 meant extremely high-severity deformity.

The subject member of the sub-center followed up with the participants by micro-mail or telephone at week 1, week 2, week 4, and week 5 after enrollment, obtaining self-healing information on CAD. At week 3 (day 15 of enrollment), and week 6 (day 42 of enrollment) after enrollment, the subject member collected information on self-healing, scores of deformity, etc. by CRF through face-to-face follow-up visits.

### Statistical methods

First, we checked the data for abnormal values and missing values, and reviewed and corrected them. Meanwhile, an otolaryngologist, who was not involved in the study, reviewed all the collected photographs and modified the scores of deformity accordingly. Since the number of prominent ear, hidden and conchal crus ear was small, they were combined into the "other types". Meanwhile, according to the standard of "Expert Consensus" [1], lop ear and cup ear were combined into constricted ear. Therefore, in the subsequent statistical analysis, the types of CAD in our study included five types: Stahl’s ear, helical rim deformity, constricted ear, compound deformity and "other types".

### Outcome indicators

1. A score of deformity was the main consequence. The VAS scale was used for evaluation. The higher the score, the higher the severity of the deformity.
2. Improvement rate was secondary consequence. First, we calculated the improvement value. Improvement value = |(score of deformity at follow-up visits—score of deformity at baseline) |/ score of deformity at baseline. The improvement was graded as excellent, good and poor based on the improvement value (**Table 1**). Then the overall improvement rate = the sum of excellent and good ears divided by the total number of ears, then multiplied by 100%.

**Table 1:** Grading of deformity improvement after self-healing of congenital auricular deformity.

Grade	Improvement rate
Excellent	The improvement rate was equal or greater than 80%
Good	The improvement rate was 50% to 79%
Poor	The improvement rate was less than 50%

### Statistical analysis

Quantitative information was described in terms of mean standard deviation ($\bar{x}\pm s$) or median and interquartile range [*M* (*IQR*)]. Qualitative information was described in terms of rate or percentage. A two-level model with repeated measures was used to compare changes in scores of deformity before and after follow-up, and covariates such as gender, type of deformity, and score of deformity at baseline were included to explore potential factors affecting the self-correction of CAD. When exploring the effects of morphological type, the Stahl’s ear was setted as a reference. The *Wald* test was used to do hypothesis testing of fixed effects parameter estimates in the repeated measures model. The *Chi-square* test was used to test the improvement rate, followed by the *Bonferroni* method to correct for further two-by-two comparison. Intention-To-Treat (ITT) and Per-Protocol (PP) analysis was conducted for scores of deformity and its influencing factors, improvement rate, respectively. The ITT analysis used a full analysis dataset. Participants without any follow-up data were excluded from PP analysis. For data without follow-up data, the previous data was used to fill in. The baseline information between the lost follow-up group (without any follow-up data) and the follow-up group (having at least one follow-up data) was further compared to better assess the impact of lost follow-up on the outcomes. P < 0.05 was considered statistically significant.

## Results

### Demographic characteristics of participants

On the basis of research funding and meeting the minimum effective sample size, a total of 135 participants was recruited into our study. **Table 2** showed 50.37% of participants were boys (with 117 ears of CAD) and 49.63% of participants were girls (with 120 ears of CAD). The mode of birth was predominantly vaginal, accounting for 61.48%.

**Table 2:** Basic information of the participants.

Category	[n(%)/($\bar{x}\pm s$)]/[*M*(*IQR*)]
No. of patients	135(100)
Total no. of ears	237(100)
Sex	
Male	68(50.37)
Female	67(49.63)
Mode of delivery	
Vaginal delivery	83(61.48)
Cesarean section	52(38.52)
Birth weight (kg)	3.36±0.38
Birth length (cm)	50.16 ±1.39
Gestational week of delivery (w)	39.31±0.93
Score of deformity at baseline[*M* (*IQR*)]	4.00 (3.00)

**Fig 1:**
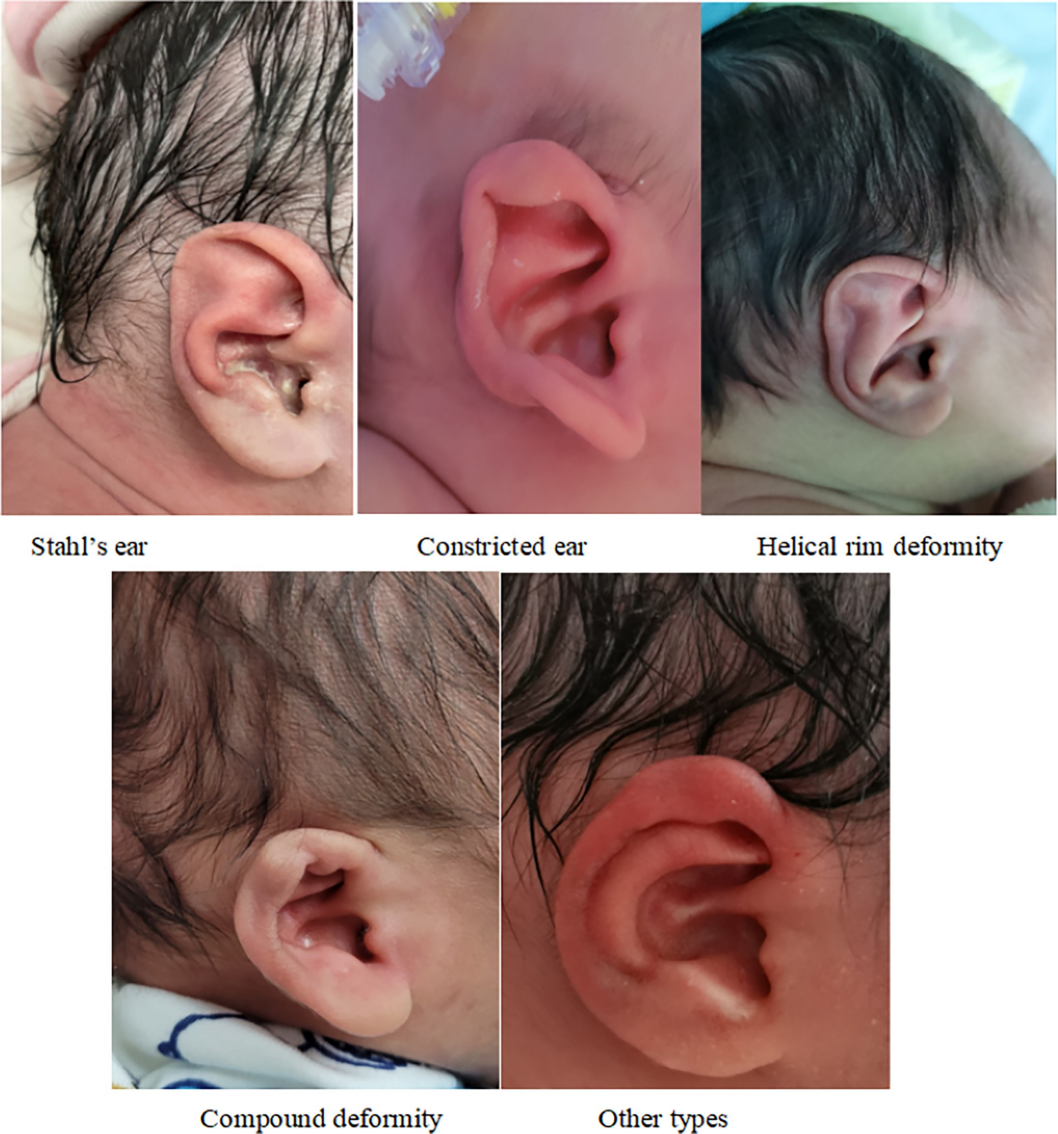
Morphological types of congenital auricular deformity.

A total of 237 ears with CAD was recruited. 15 participants (6.33%) were left ear malformations only, 18 participants (7.59%) were right ear malformations only, and 102 participants (86.08%) were binaural malformations. **Fig 1** showed different types of CAD. The top morphological type was constricted ear (107 ears, 45.15%), followed by helical rim deformity (71 ears, 29.86%). Stahl’s ear, compound deformity and "other types" had similar percentages.

Stahl’s ear, constricted ear, helical rim deformity, compound deformity and "other types" were included in our study at baseline.

ALL the participants (237ears) were included into ITT analysis. Twenty-nine participants without any follow-up data were excluded from the PP analysis.

### Score of deformity

In the ITT analysis, the score of deformity at baseline, week 3 and week 6 after enrollment was 4.00 (*IQR*: 3.00), 3.00 (*IQR*: 3.00) and 2.00 (*IQR*: 3.00). The results from PP populations were similar except for a slight difference in *IQR* at week 6 (**Table 3**).

**Table 3:** Deformity scores for different types of congenital auricular deformity at different time.

Morphological type	Baseline [*M*(*IQR*)]	Week 3[*M(IQR*)]		Week 6[*M(IQR)*]	
		ITT	PP	ITT	PP
Total	4.00(3.00)	3.00(3.00)	3.00(3.00)	2.00(4.00)	2.00(3.00)
Stahl’s ear	3.00(3.00)	0.00(1.00)	0.00(2.00)	0.00(0.00)	0.00(0.00)
Other types	2.00(3.00)	2.50(3.00)	3.00(4.50)	2.50(5.00)	3.00(4.50)
Helical rim deformity	4.00(2.00)	3.00(2.00)	3.00(2.00)	2.00(3.00)	2.00(3.00)
Constricted ear	4.00(3.00)	3.00(3.00)	3.00(3.00)	2.00(4.00)	2.00(4.00)
Compound deformity	6.00(3.00)	4.00(5.00)	4.00(5.00)	3.00(5.00)	3.00(4.00)

**Fig 2:**
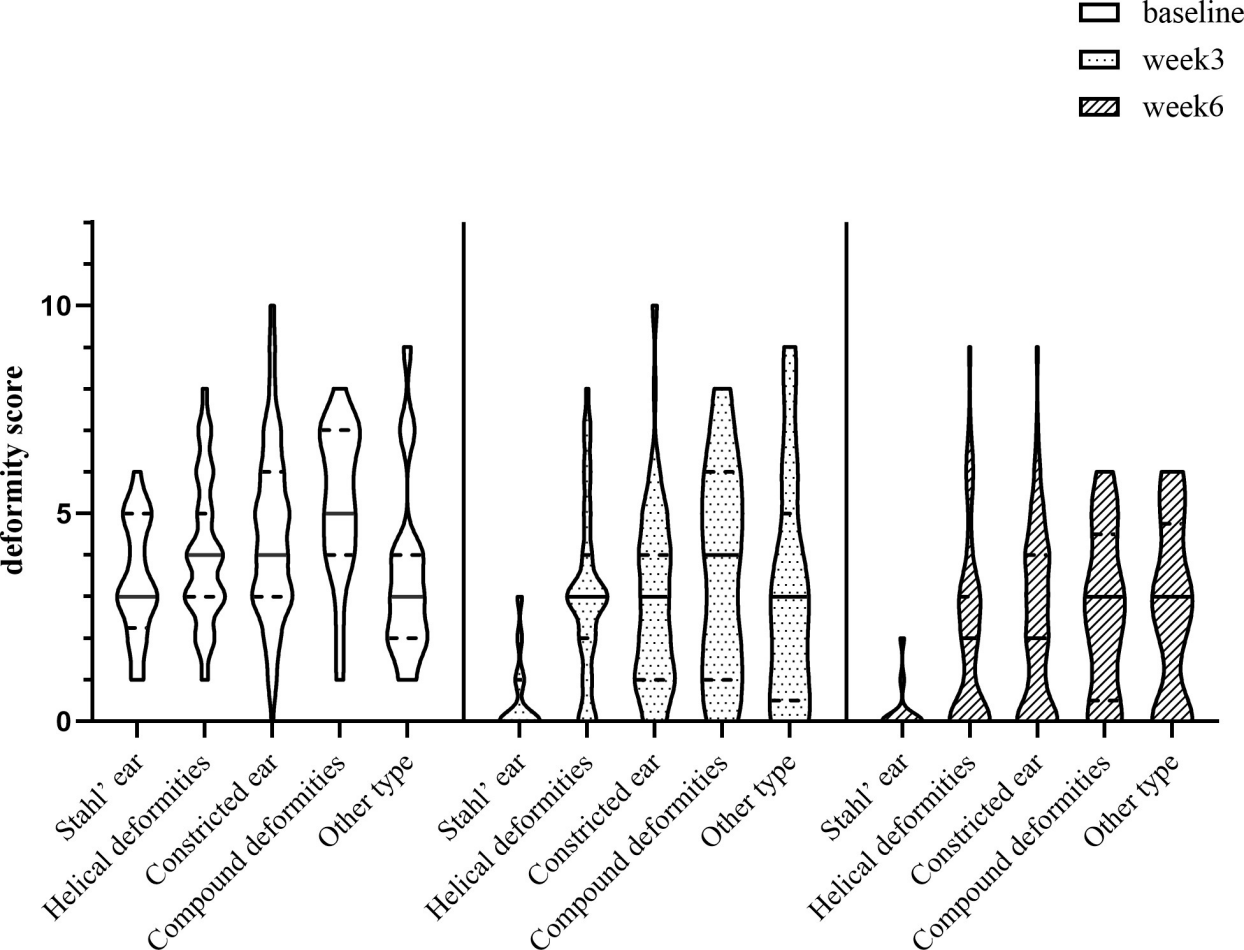
The violin distribution of deformity scores for the five morphological types at follow-up time points.

In terms of morphological types, the ITT analysis showed that the score of deformity of Stahl’s ear, “other types”, helical rim deformity, constricted ear and compound deformity was 0.00 (*IQR*: 1.00), 2.50 (*IQR*: 3.00), 3.00 (*IQR*: 2.00), 3.00 (*IQR*: 3.00) and 4.00 (*IQR*: 5.00) at week 3 after enrollment. And that was 0.00 (*IQR*: 0.00), 2.50 (*IQR*: 5.00), 2.00 (*IQR*: 3.00), 2.00 (*IQR*: 4.00) and 3.00 (*IQR*: 5.00) at week 6 (**Table 3**, **Fig 2**).

The solid black line in the violin plot represented the median scores of deformity. As shown in the figure, the solid black line of the five morphology types shifted downward to varying degrees from baseline to postnatal week 6, indicating a decrease in scores of deformity.

**Fig 3:**
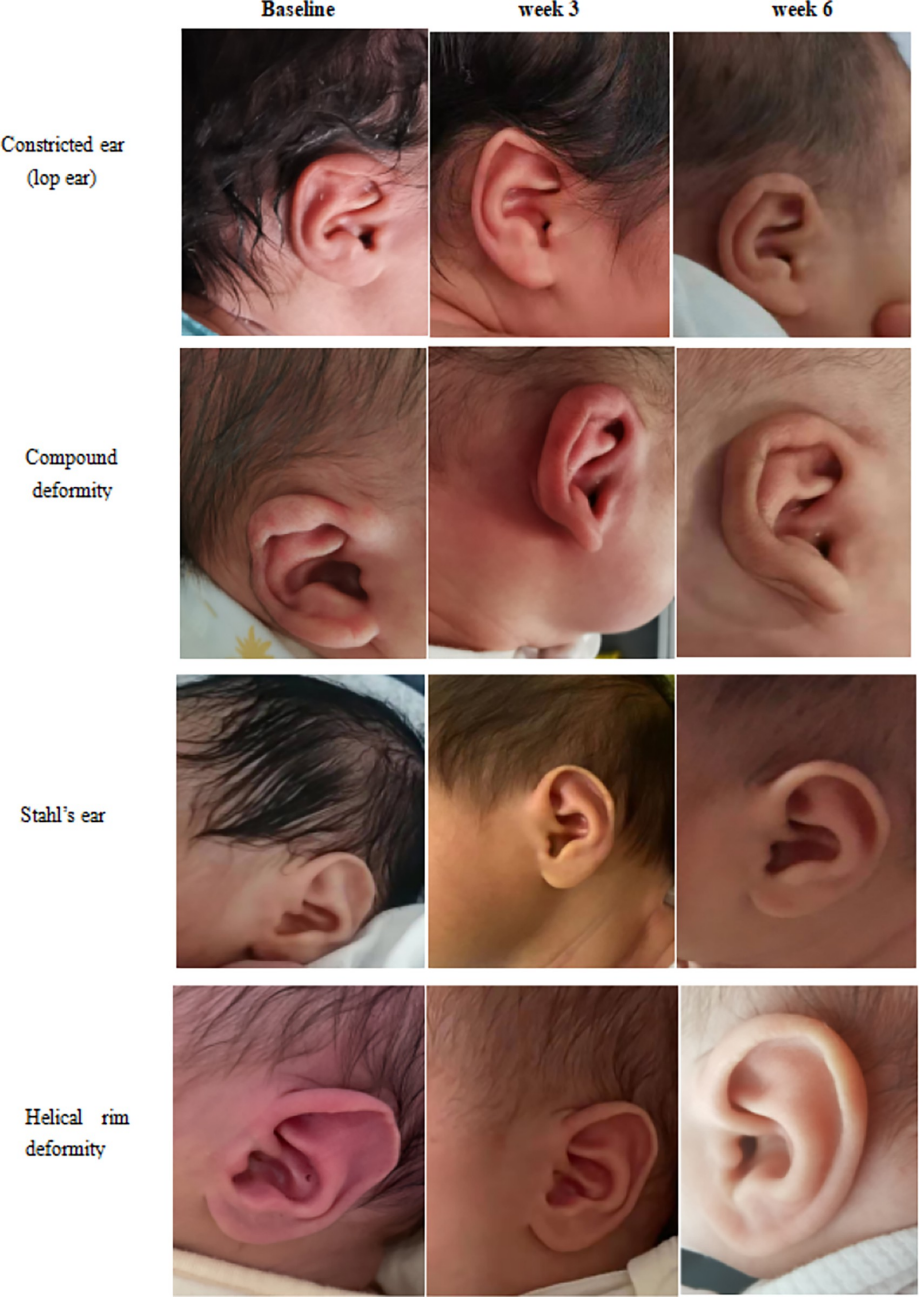
Photographs of self-correcting changes in some morphological types at follow-up time points.

In the PP analysis, the score of deformity of "other types" was 3.00 (*IQR*: 4.50) at week 3 and week 6, respectively. These were different from the results of ITT analysis, while others were basically the same (**Table 3** and **Fig 2**). **Fig 3** showed the changes in auricular morphology before and after the follow-up of some CAD.

### Two-level repeated measures model

**Table 4A**, **model B** from ITT analysis and **Table 4B**, **model E** from PP analysis both showed that the coefficients of the time were signific ant (*χ*^2^ = 278.256, P < 0.001; *χ*^2^ = 308.37, P < 0.001), indicating a decreasing trend in scores of deformity over time.

**Table 4:** a. A two-level analytical model of factors associated with self-correction affecting congenital auricular deformity (ITT-analysis). b. A two-level analytical model of factors associated with self correction affecting congenital auricular deformity (PP-analysis).

Effects	Parameters	Coefficient (Estimation error)		
		Model A	Model B	Model C
Fixed effects	*β*_0_	3.247(0.121)*	4.206(0.133)*	3.088(0.332)*
	*β*_time_		-1.151(0.069)*	-1.158(0.063)*
	*β*_sex_			0.091(0.139)
	*β*_score of deformity at baseline_			0.782(0.036)*
	*β*_helical rim deformity_			1.005(0.269)*
	*β*_constricted ear_			0.992(0.261)*
	*β*_compound deformity_			1.205(0.342)*
	*β*_other types_			1.264(0.333)*
Random effects				
Level 2	$\sigma_{u0}^{2}$	2.142(0.332)*	2.674(0.321)*	0.465(0.110)*
Level 1	$\sigma_{e0}^{2}$	3.089(0.232)*	1.724(0.131)*	1.536(0.115)*
-2 *LGLS*		2559.763	2351.409	2048.073*
Effects	Parameters	Coefficient (Estimation error)		
		Model D	Model E	Model F
Fixed effects	*β*_0_	4.248(0.142)*	4.404(0.157)*	3.169(0.369)*
	*β*_time_		-1.159(0.066)*	-1.156(0.065)*
	*β*_sex_			0.064(0.155)
	*β*_score of deformity at baseline_			1.476(0.081)*
	*β*_helical rim deformity_			1.130(0.307)*
	*β*_constricted ear_			1.103(0.269)*
	*β*_compound deformity_			1.414(0.316)*
	*β*_other types_			1.283(0.368)*
Random effects				
Level 2	$\sigma_{u0}^{2}$	2.265(0.387)*	3.082(0.386)*	0.645(0.128)*
Level 1	$\sigma_{e0}^{2}$	2.918(0.210)	1.610(0.116)*	1.589(0.115)*
-2 *LGLS*		2427.976	2201.022	1995.939

Note: (1) “*”meant P < 0.05. (2) Level 2 was “ear”. Level 1 was “time”. (3) “-2 *LGLS*” meant -2log-likelihood value.

**Table 4A**, **model C** showed that the coefficient for the score of deformity at baseline had statistically significant (*χ*^2^ = 471.845, P < 0.001). That was, for every 1-point increase in the score of deformity at baseline, the score of deformity increased by 0.782 points after follow-up. The coefficients for helical rim deformity (*χ*^2^ = 13.957, P < 0.001), constricted ear (*χ*^2^ = 14.447, P < 0.001), compound deformity (*χ*^2^ = 12.411, P < 0.001) and "other types" (*χ*^2^ = 14.410, P < 0.001) were statistically significant, indicating that the scores of deformity each of these four morphological sub-types were higher than that for Stahl’s ear after follow-up. We found no significant differences among the scores of deformity in different genders (*χ*^2^ = 0.492, P = 0.483). Results from PP analysis in **Table 4B**, **model F** gave similar with results from ITT analysis in **Table 4A**, **model C**, but the absolute value of the coefficient was large.

### The improvement rate

From the ITT analysis, the total improvement rate of CAD at week 3 was 29.96% (71/237 ears), with the highest to lowest improvement rate being Stahl’s ear (55.00%), “other types” (38.89%), constricted ear (27.62%), compound deformity (26.09%) and helical rim deformity (25.35%) (**Table 5)**. From the PP analysis, the total improvement rate of CAD at week 3 was 38.50% (72/187 ears), with the highest to lowest improvement rate being Stahl’s ear (75.00%), compound deformity (40.00%), “other types” (35.29%), helical rim deformity (34.55%), and constricted ear (34.52%) (**Table 5)**.

**Table 5:** The improvement rates of different types of congenital auricular deformity at week 3 and week 6 after birth.

Morphological type	week 3(%)		week 6 (%)	
	ITT	PP	ITT	PP
Stahl’s ear	55.00^#^	75.00^&^	60.00^#^	87.50^&^
Other types	33.33	35.29	38.89	41.18
Constricted ear	27.62	34.52	32.38	45.24
Compound deformity	26.09	40.00	30.43	46.67
Helical rim deformity	25.35	34.55	40.85	56.36
*χ* ^2^	13.799	9.614	11.518	11.002
*P*	0.008	0.047	0.021	0.027

Note

“^#^” meant that stahl’s ear ear *vs* other types, constricted ear, compound deformity and helical rim deformity, P < 0.05 in the ITT analysis.

“^&^” meant that stahl’s ear ear *vs* other types, constricted ear, compound deformity and helical rim deformity, P < 0.05 in the PP analysis.

In the ITT analysis, the total improvement rate of CAD at week 6 was 37.13% (88/237), with the highest to lowest improvement rate being Stahl’s ear (60.00%), helical rim deformity (40.85%), “other types” (38.89%), constricted ear (32.38%), and compound deformity (30.43%). However, in the PP analysis, the total improvement rate of CAD at week 6 was 51.87% (97/187), with the highest to lowest improvement rate being Stahl’s ear (87.50%), helical rim deformity (56.36%), compound deformity (46.67%), constricted ear (45.24%), and “other types” (41.18%). Subsequently, we performed a two-by-two comparison within the group. **Table 5** showed that the improvement rate of the Stahl’s ear at week 3 and week 6 after enrollment was higher than that of four other morphological types (P < 0.05), while none of significant differences between the helical rim deformity, constricted ear, compound deformity, and "other types" were found (P > 0.05). Results from PP analysis and ITT analysis gave the same results.

The comparison of baseline characteristics between the lost follow-up group and the follow-up group was further conducted. **Table 6** showed that differences between the two groups in baseline characteristics (such as age, gender, score of deformity, and morphological types, etc.) were not statistically significant.

**Table 6:** Comparison of baseline characteristics between the lost follow-up group and the non lost group.

Category	Lost to follow-up group [%/($\bar{x}\pm s$)//M*(IQR*)]	Follow-up group [%/($\bar{x}\pm s$)//*M*(*IQR*)]	*t*/*χ*^2^/Z	*P*
Sex			0.027	0.869
Male	15	53		
Female	14	53		
Mode of delivery			1.864	0.172
Vaginal delivery	21	62		
Cesarean section	8	44		
Birth length (cm)	50.48±1.66	50.07±1.30	2.50	0.12
Birth weight (kg)	3.44±0.40	3.34±0.37	0.74	0.13
Gestational week of delivery (w)	39.53±1.11	42.55±3.98	0.83	0.36
Score of deformity at baseline[*M (IQR*)]	3.00(3.00)	4.00(3.00)	2.28	0.05
Morphological type			3.505	0.477
Stahl’s ear	5	16		
Other types	1	17		
Helical rim deformity	16	55		
Constricted ear	22	84		
Compound deformity	6	15		

## Discussion

This study found that the scores of deformity at week 3 and week 6 were 3.00 and 2.00, respectively, which were lower than that at baseline. Also, the coefficient of the variable “time” in the repeated measures model was negative, indicating an overall decreasing trend in the scores of deformity. That was, CAD had a tendency to heal itself. This was consistent with the findings obtained from previous studies [8,16].

This study showed the overall improvement rate of CAD was 29.96% and 37.13% at week 3 and 6, respectively. This result was dissimilar from the study by Byrd (33%) [1] and also lower than the study by Wang (40.77%) [12]. Possible reasons for the differences between the results of the studies were: (1) different lengths of follow-up. The participants in our study were followed up until 6 weeks after birth, whereas Byrd’s and Wang’s follow-up was conducted on the 7th day and at 1 month after birth, respectively, which may have contributed to the discrepancy in the results. It has been established that the ability of the auricular cartilage to maintain a certain level of tone and malleability is closely related to its hyaluronic acid content and alignment within the cartilage [17]. Higher levels of hyaluronic acid indicate better ductility and plasticity of the auricle. Hyaluronic acid is positively correlated with high estrogen levels [18,19]. Estrogen, of maternal origin, peaks on the third day after birth, then declines and returns to normal levels by the sixth week of life, which is the current mechanism for non invasive correction of CAD. However, self-healing mechanism of CAD is not yet clear. (2) The difference in the morphological types of deformities included. Our study was dominated by constricted ear and helical rim deformity, while Byrd’s study was dominated by prominent ear and lop ear, and Wang’s study was dominated by lop ear, which may have influenced the overall self-correction of CAD. The results of PP analysis still reflected the self-healing trend of CAD. However, the high and low rankings of improvement rates for different morphological types of CAD differed from the results of the ITT analysis. This was associated with the exclusion of 29 participants without any follow-up data. Given the importance of the timing of early intervention in CAD, we believed it was more appropriate to use the results of the ITT analysis as a reference. Although van observed 1000 Japanese babies from birth to 1 year and found that the prevalence of lop ears decreased from 38.1% at birth to 6.1% at 1 year of age [16]. And the study by Kim demonstrated that the self-healing rate of CAD was as high as 50% 1 year after birth [13], suggesting a tendency for CAD to heal itself up to 1 year of age. But the results from our study showed that the self-correction of CAD in the short term was not high. Based on the changing patterns of estrogen and hyaluronic acid in newborns, combined with the results of this study, it seems to indicate that most children with CAD still have a need for early non-invasive correction.

Few previous studies have examined the factors influencing the self-correction of CAD. We fitted two-level repeated measure models for ITT analysis and PP analysis, respectively, and reached consistent conclusions. The repeated measure model in this study showed that the score of deformity in Stahl’s ear was lower than those of four other morphological types, respectively. In terms of improvement rate, there were differences between the morphological types at week 3 and week 6 of follow-up, indicating morphological type was one of the self-correcting influencing factors of CAD, which was consistent with our research hypothesis and previous studies [10]. This study showed that the improvement rates of Stahl’s ear at week 3 and week 6 were both higher than other four morphological types, respectively, indicating Stahl’s ear had a better tendency to self-correct. A similar result was observed in the study of Wang [12]. According to the pattern of estrogen and hyaluronic acid changes in newborns, combined with the results of this study, it was recommended that children diagnosed as Stahl’s ear should be observed until 1 week after birth and then decide whether to perform ear mold correction based on its self-recovery. Nevertheless, Kim’s study showed that self-correction of helical rim deformity was better [13], while van concluded that self-correction of lop ear was better [16], suggesting that there may be racial differences in self-correction rates of morphological types. Notably, we observed that only one prominent ear improved at the 6-week follow-up, while the others did not, suggesting that the self-correction of prominent was poor. Pevious study has confirmed that the prevalence of prominent ear increased with age and may be related to continuous pressure on the auricle during lateral sleep at night [20]. Considering that newborns’ ears are basically set by the age of 1 year, clinicians and guardians require determining the possibility of early correction of prominent ear.

This study revealed that score of deformity at baseline was also one of the factors affecting self-correction of CAD, consisting with our research hypothesis. The higher score of deformity at baseline (the greater severe the deformity), the less effective the self-correction. As the severity of auricular deformity increases, the degree of muscle abnormalities, morphological distortions, and even muscle developmental abnormalities in the CAD affected ear increase, making self-correction of the ear alone difficult to obtain a better recovery, and such deformities may require the intervention of ear molds given at an early stage. Therefore, it is necessary and timely for clinicians to determine the severity of the deformity after screening for CAD to better judge the timing of ear mold intervention. Although previous studies have shown gender differences in the occurrence of CAD, and it was thought that differences in hormone levels may be one of the mechanisms responsible for the differences in onset. However, unlike our research hypothesis, gender was not found to have an effect on the self-correction of CAD.

The limitations of this study were as follows: First, the rate of missed visits (by ears) was high. The rate of missed visit was 28.15% and 34.81% at week 3 and week 6, respectively. The reasons for missing visits were partly due to the parents putting ear molds on their children themselves, and partly because the parents thought that their child’s auricular deformity was not serious, thus did not want to continue the study. The results of the study may have been slightly distorted due to this. Second, the sample size of some morphological types of CAD, such as prominent ear, hidden ear and conchal crus, was small, and the self-correction of these morphological types could not be analyzed separately.

## Conclusions

Overall, this prospective study showed that CAD had a tendency to self-correction in the short term, with self-correction rates of 29.96% and 37.13% at week 3 and 6 after birth, respectively. Morphological types and severity of deformity are the main influencing factors influencing CAD’ self-correction. Stahl’s ear has a better self-correction, and can be observed for a period of time before deciding whether to give ear mold intervention. However, the overall rate of self-healing in other morphologic subtypes is not high. Early non-invasive correction should be considered for most CAD according to our findings.The greater severity of deformity, the worse the self-correction effect, and for those with a high severity of deformity, ear mold needs to be implemented as soon as possible. We recommend that those patients with a high degree of deformity or a low degree of self-healing should receive non-invasive correction within 6 weeks after birth. Therefor, clinicians must be well informed in order to make sound clinical decisions early on.

## Supporting information

S1 Data set The minimal data set underlying the findings in our manuscript. (XLSX)
